# A Novel Role for *DNA Methyltransferase 1* in Regulating Oocyte Cytoplasmic Maturation in Pigs

**DOI:** 10.1371/journal.pone.0127512

**Published:** 2015-05-26

**Authors:** Yanjun Huan, Bingteng Xie, Shichao Liu, Qingran Kong, Zhonghua Liu

**Affiliations:** 1 College of Life Science, Northeast Agricultural University, Harbin, Heilongjiang Province, China; 2 Dairy Cattle Research Center, Shandong Academy of Agricultural Sciences, Jinan, Shandong Province, China; Qingdao Agricultural University, CHINA

## Abstract

Maternal factors are required for oocyte maturation and embryo development. To better understand the role of *DNA methyltransferase 1* (*Dnmt1*) in oocyte maturation and embryo development, small interfering RNA (siRNA) was conducted in porcine oocytes. In this study, our results showed that *Dnmt1* localized in oocyte cytoplasm and its expression displayed no obvious change during oocyte maturation. When siRNAs targeting *Dnmt1* were injected into germinal vesicle (GV) stage oocytes, *Dnmt1* transcripts significantly decreased in matured oocytes (P<0.05). After *Dnmt1* knockdown in GV stage oocytes, the significant reduction of glutathione content, mitochondrial DNA copy number, glucose-6-phosphate dehydrogenase activity and expression profiles of maternal factors and the severely disrupted distribution of cortical granules were observed in MII stage oocytes (P<0.05), leading to the impaired oocyte cytoplasm. Further study displayed that *Dnmt1* knockdown in GV stage oocytes significantly reduced the development of early embryos generated through parthenogenetic activation, *in vitro* fertilization and somatic cell nuclear transfer (P<0.05). In conclusion, *Dnmt1* was indispensable for oocyte cytoplasmic maturation, providing a novel role for *Dnmt1* in the regulation of oocyte maturation.

## Introduction

The ability of oocytes to support the subsequent embryonic development involves nuclear and cytoplasmic maturation [1, 2]. Oocyte nuclear maturation refers to the dynamics of chromosome separation, while cytoplasmic maturation involves the redistribution of cytoplasmic organelles and the storage and progressing of mRNA and proteins, and is the core factor for achieving the subsequent embryonic development [3, 4].

Generally, oocyte cytoplasmic maturation is a complex progress and affected by multiple factors [1]. The quality of oocyte cytoplasmic maturation is closely correlated with glutathione (GSH) content, mitochondrial DNA (mtDNA) copy number and cortical granule (CG) distribution, etc. [2, 5, 6], and the stored maternal factors also take a key role in oocyte cytoplasmic maturation [2, 7].

*Dnmt1* is a maternal factor, and could be indispensable for oocyte maturation and the subsequent embryonic development [8, 9]. It is known that *Dnmt1* gene locus encodes three isoforms by alternative usage of multiple first exons: *Dnmt1s* (somatic form), *Dnmt1o* (oocyte specific form) and *Dnmt1p* (the form in pachytene spermatocytes) [10]. Among these isoforms, *Dnmt1o*, a truncated version, lacking the first 118 amino acids at the amino terminus present in *Dnmt1s*, is the only known form of maintenance methyltransferase before zygotic genome activation (ZGA), as *Dnmt1s* is zygotic origin and *Dnmt1p* is an inactive form, suggesting that *Dnmt1o* is the sole maternal maintenance methyltransferases [11], essential for epigenetic reprogramming and embryo development [8, 9]. Previous studies have shown that loss of *Dnmt1o* in oocytes impairs the maintenance of DNA methylation imprints and embryo development, while *Dnmt1* downregulation in cloned embryos improves early embryonic development, indicating that *Dnmt1o* is necessary for oocyte maturation [8, 11–13]. *Dnmt1o* has been shown to be present in the cytoplasm of oocytes and preimplantation embryos [11, 14, 15], and could regulate mitochondrial function and gene expression and protect cells from oxidative stress [16–18]. Thus, *Dnmt1* may regulate oocyte cytoplasmic maturation, thereby determining the subsequent embryonic development. However, the role of *Dnmt1* in oocyte maturation and the subsequent embryonic development remains unclear.

In this study, RNA interference was employed to investigate the role of *Dnmt1* in oocyte maturation and embryo development. After siRNAs targeting *Dnmt1* were injected into porcine GV stage oocytes, *Dnmt1* transcripts significantly decreased and oocyte cytoplasmic maturation was severely impaired. Furthermore, *Dnmt1* knockdown in GV stage oocytes significantly reduced the development of early embryos. These results demonstrated that *Dnmt1* was required for oocyte cytoplasmic maturation, determining the subsequent embryonic development. This work revealed a novel role for *Dnmt1* in the regulation of oocyte cytoplasmic maturation, and would have important implications in oocyte maturation and embryo development.

## Materials and Methods

Chemicals were purchased from Sigma Aldrich Corporation (St. Louis, MO, USA), and disposable and sterile plasticware was obtained from Nunclon (Roskilde, Denmark), unless otherwise stated.

All experiments were approved by the Animal Care Commission of Northeast Agriculture University, according to animal welfare laws, guidelines and policies. And, all surgery was performed under sodium pentobarbital anesthesia, and all efforts were made to minimize suffering.

### Donor cell collection and culture

Donor cell culture has been described previously [19]. Briefly, porcine fetuses were obtained from a sow at day 35 of pregnancy after the sow was anaesthetized and sacrificed, then porcine fetal fibroblasts (PFFs) were isolated from 35-day-old fetuses under sodium pentobarbital anaesthesia. After removal of fetal head, internal organs and limbs, the remaining tissues were finely minced into pieces, digested with 0.25% trypsin-0.04% ethylenediaminetetraacetic acid solution (GIBCO), and then dispersed in high glucose enriched Dulbecco’s modified Eagle’s medium (DMEM, GIBCO) containing 10% fetal bovine serum (FBS, GIBCO) and 1% penicillin-streptomycin (GIBCO). The dispersed cells were centrifuged, resuspended and cultured in DMEM. Until confluence, PFFs were digested, centrifuged, resuspended in FBS containing 10% dimethyl sulfoxide and stored in liquid nitrogen until use. Prior to SCNT, PFFs were thawed, cultured and subsequently used in 3–5 passages.

### Oocyte collection and *in vitro* maturation (IVM)

IVM of denuded oocytes has been described in our previous study [20]. Briefly, porcine ovaries were collected from a slaughterhouse of Harbin Dazhong Roulian Food Co., Ltd., located in Harbin city, Heilongjiang province. Just after ovary exposure, they were placed in physiological saline with antibiotics at 37°C and transported to the laboratory. Follicles were aspirated, and follicular contents were washed with HEPES buffered Tyrode's lactate. Cumulus-oocyte complexes were recovered, and cumulus cells were removed with hyaluronidase. Then, denuded oocytes were washed and cocultured with mural granulosa cells in maturation medium. After 42 h, oocytes were vortexed in hyaluronidase for 30 sec. Only oocytes with a visible polar body, regular morphology and homogenous cytoplasm were used in the subsequent experiments.

### siRNA design, synthesis and microinjection

According to the requirement of Invitrogen Block-iT RNAi Designer and the information of *Dnmt1* mRNA sequence, three Stealth siRNAs related to *Dnmt1* conserved domains including the replication foci domain (RFD), bromo adjacent homology domain (BAH) and cytosine-C5 specific DNA methylase domain (DCM) were designed and synthesized (Invitrogen), and the sequences were as following: siRNA-RFD: CCCGTCTCTTGAAGGTGGTGTTAAT, siRNA-BAH: CATAGCAAAGTGAAGGTCATCTATA and siRNA-DCM: GATAAGAAGTTTGTCAGCAACATCA. Then, siRNAs were dissolved with Rnase free H_2_O to the concentration at 20 μM and microinjected into GV stage oocytes in 200 μl drop of manipulation medium supplemented with 7.5 μg ml^-1^ cytochalasin B and bovine serum albumin (BSA) using Sterile Femtotips and the FemtoJet express microinjector (Eppendorf) [11]. The injection condition was 250 hpa Injection Pressure, 60 hpa Comensation Pressure and 0.7 sec Injection Time, and approximate 10 pl siRNAs were injected into each oocyte. The same amount of negative siRNAs or Rnase free water was injected as the control, and FITC labeled nonsilencing siRNA was used to evaluate the successful rate of injection. Immediately after microinjection, oocytes were washed and cocultured with mural granulosa cells in maturation medium.

### Immunofluorescence

Immunofluorescence has been described previously [11]. Briefly, oocytes were treated with acidic Tyrode's solution to remove zona pellucida, fixed with 4% paraformaldehyde for 30 min, permeabilized in 1% Triton X 100 and blocked in 1% BSA supplemented DPBS for 1 h. Then, oocytes were incubated in anti-Dnmt1 antibody (Santa Cruz) or anti-beta tubulin antibody at 4°C overnight. After three washes with DPBS containing 0.1% Tween 20 and 0.01% Triton X 100, oocytes were labeled with FITC conjugated secondary antibody (Santa Cruz) for 1 h at room temperature. Then, oocytes were stained with 10 μg ml^-1^ PI for 5 min in the dark. After staining, oocytes were washed, mounted on slides and examined under a fluorescence microscope.

### Intracellular GSH content

Intracellular GSH content has been measured in previous studies [21, 22]. Briefly, for staining, matured oocytes were stained in 5 μM Cell Tracker Blue CMF_2_HC (Invitrogen) for 30 min. After staining, oocytes were incubated in manipulation medium for another 30 min and washed three times with DPBS. Then, oocytes were observed and photographed under a fluorescence microscope, and the fluorescence intensities were analyzed using Image Pro Plus. For measurement, GSH content in oocytes was determined using a GSH assay kit (Beyotime) according to the manufacturer’s instruction. After samples were frozen and thawed three times using liquid nitrogen and 37°C water, GSH content was measured by the 5,5’-dithiobis (2-nitrobenzoic acid) (DTNB) GSSG reductase recycling assay. According to the standard curve, total GSH amounts in samples were calculated and divided by the number of oocytes to get GSH concentration per oocyte.

### Determination of G6PDH activity

Glucose-6-phosphate dehydrogenase activity (G6PDH) activity was detected by brilliant cresyl blue (BCB) staining [20]. Briefly, oocytes were washed in manipulation medium and stained in 13 μM BCB for 90 min. After BCB incubation, oocytes were washed three times in DPBS and photographed under a fluorescence microscope. Then, according to the cytoplasmic coloration, oocytes with blue cytoplasm (BCB positive) were calculated.

### CG distribution

The evaluation of CG distribution has been reported [23]. Briefly, oocytes were fixed with 4% paraformaldehyde for 30 min and washed in DPBS containing 0.3% BSA. Then, after treatment with 0.1% Triton X 100, oocytes were washed and incubated in 100 mg L^-1^ FITC conjugated peanut for 30 min in the dark. Finally, oocytes were washed and mounted on slides. Then, CG distribution were examined under a fluorescence microscope.

### Mitochondrial staining and mtDNA copy number

Mitochondrial staining and the calculation of mtDNA copy number have been described [6, 23]. Briefly, for staining, oocytes were incubated in 200 nM Mito-Tracker Green (Molecular Probes) for 30 min. After incubation, oocytes were washed in manipulation medium and observed under a fluorescence microscope. The fluorescence intensities were analyzed using Image Pro Plus. For measurement, absolute quantitative real time PCR was applied. The target fragment of porcine mtDNA (GenBank accession no. NC_000845) was amplified by PCR using the primers (forward, 5’-TCGGAACAGACCTCGTAGAATG-3’ and reverse, 5’-GGTAATGATGAATGGCAGGATAAA-3’). Then, the amplified fragments were connected with T vectors, transformed and sequenced. The concentration of the successfully recombinant vector was detected and serially diluted to 5×10^2^–10^7^ copy μL^-1^ as the standard samples. The cycle threshold (CT) values of the standard samples and every matured oocyte were determined with quantitative real time PCR. Then, the standard curve based on the relationship between CT values and mtDNA copy numbers was drawn, and the mtDNA copy number per oocyte was calculated.

### Quantitative real time PCR

Measurement of gene expression in oocytes with quantitative real time PCR has been reported in our previous studies [11, 19]. Briefly, total RNA was extracted from 30 pooled oocytes using an RNeasy Mini Kit (Qiagen) according to the manufacturer’s instruction. Reverse transcription was performed using a PrimeScriptRT Reagent Kit (TaKaRa) with the following parameters: 37°C for 15 min and 85°C for 5 sec, and the cDNA was stored at -20°C until use. For quantitative real time PCR, reactions were performed in 96-well optical reaction plates using SYBR Premix ExTaq II (TaKaRa) and a 7500 Real Time PCR System (Applied Biosystems) with the following conditions: 95°C for 30 sec, followed by 40 two-step cycles of 95°C for 5 sec and 60°C for 34 sec and finally a dissociation stage consisting of 95°C for 15 sec, 60°C for 1 min and 95°C for 15 sec. For every sample, the CT values were obtained from three replicates. The primers for the amplification of target and internal reference genes were presented in Table A in S1 File. The relative expression levels of target genes were analyzed using the 2^−ΔΔCT^ method.

### Parthenogenetic activation (PA), *In vitro* fertilization (IVF) and somatic cell nuclear transfer (SCNT), and embryo culture

After microinjection, matured oocytes were subjected to PA, IVF and SCNT [11, 19]. Briefly, for PA, oocyte activation was induced by two direct pulses of 1.2 kv cm^-1^ for 30 μs in fusion medium. For IVF, the semen was incubated, resuspended and washed in DPBS supplemented with 0.1% (w/v) BSA. The spermatozoa were diluted with modified Tris-buffered medium (mTBM) to the appropriate concentration. Matured oocytes were washed in mTBM, transferred into fertilization medium and coincubated with spermatozoa. For SCNT, matured oocytes and PFFs were placed in manipulation medium. After enucleation, donor cells were placed into the perivitelline space. Fusion and activation of the cell-cytoplast complexes were induced, and the fusion rate was confirmed by microscopic examination. Then, the activated, IVF and cloned embryos were cultured in porcine zygote medium-3 (PZM-3) for subsequent development. The cleavage and blastocyst rates of PA, IVF and SCNT embryos were evaluated at 48 h and 156 h post activation, respectively.

### Nuclear staining

Blastocysts derived from PA, IVF and SCNT were treated with acidic Tyrode's solution to remove zona pellucida, fixed with 4% paraformaldehyde for 30 min and stained in DPBS containing 10 mg L^-1^ Hoechst 33342 for 5 min in the dark. After staining, blastocysts were washed and mounted on slides. Then, blastocyst cell numbers were examined under ultraviolet light from a fluorescence microscope.

### Statistical analysis

Differences in data (mean ± SEM) were analyzed with SPSS statistical software. Statistical analysis of data regarding oocyte maturation and embryo development was performed using the general linear model (GLM). The data for gene expression was analyzed with one-way ANOVA. The t-test was applied to analyze GSH content, G6PDH activity, mtDNA copy number and CG distribution. For all analyses, differences were considered to be statistically significant when P<0.05.

## Results

### *Dnmt1* was effectively reduced in oocytes after siRNA injection

**Fig 1:**
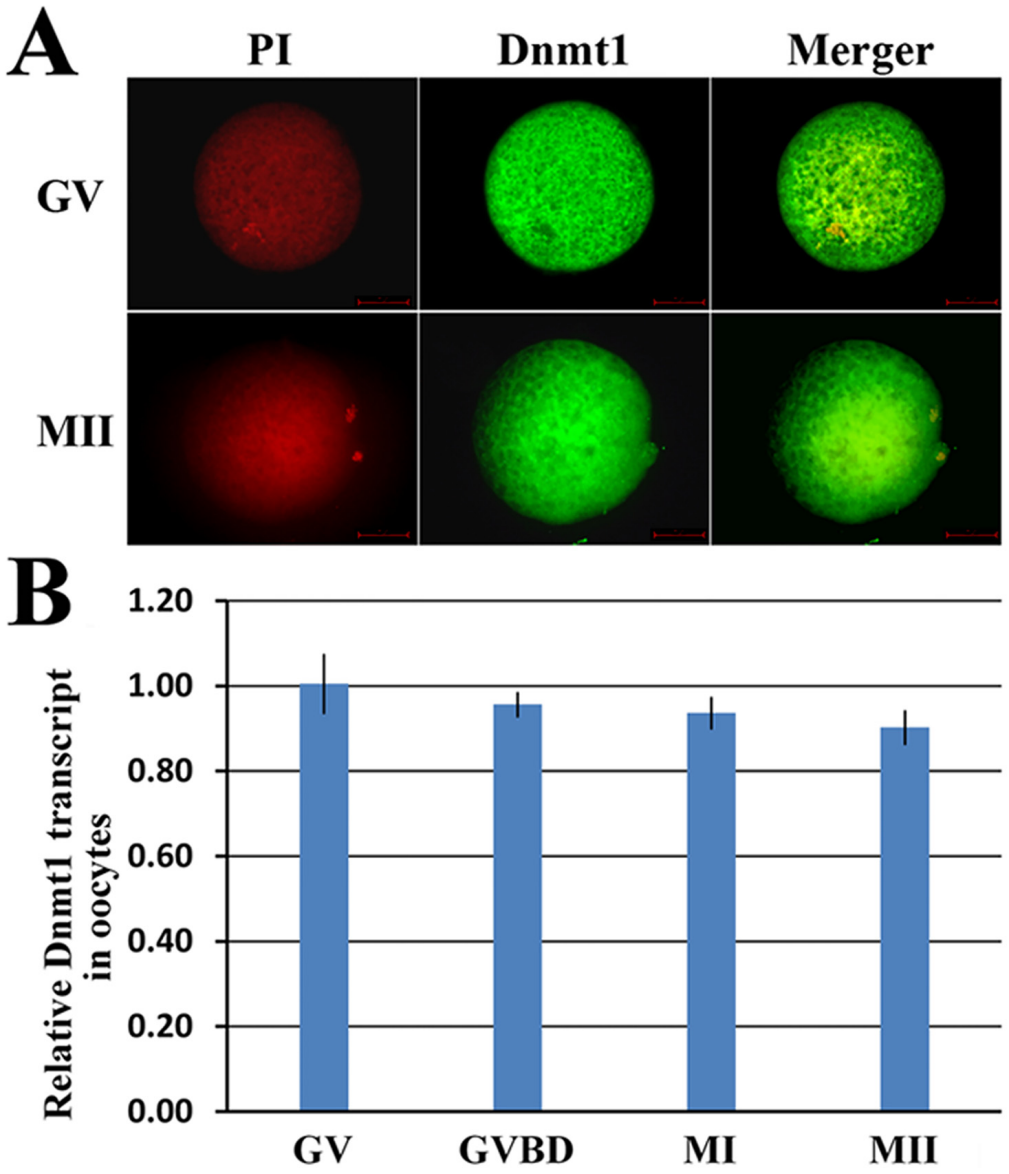
The expression and location of Dnmt1 in oocytes. A, the cytoplasmic location of Dnmt1 in GV and MII stage oocytes (×400), and B, relative Dnmt1 transcription levels during oocyte maturation. No obvious changes of Dnmt1 expression were observed in GV, GVBD, MI and MII stage oocytes. GV, GVBD, MI and MII stage oocytes were collected at 0 h, 19 h, 24 h and 42 h, respectively.

During porcine oocyte maturation, the expression pattern of *Dnmt1* was examined. Our results showed that *Dnmt1* localized in oocyte cytoplasm and its expression did not significantly change (Fig 1). And, the established systems of denuded oocyte maturation and oocyte injection did not affect *Dnmt1* expression, oocyte maturation and embryo development (S1 Fig and Table B in S1 File).

**Fig 2:**
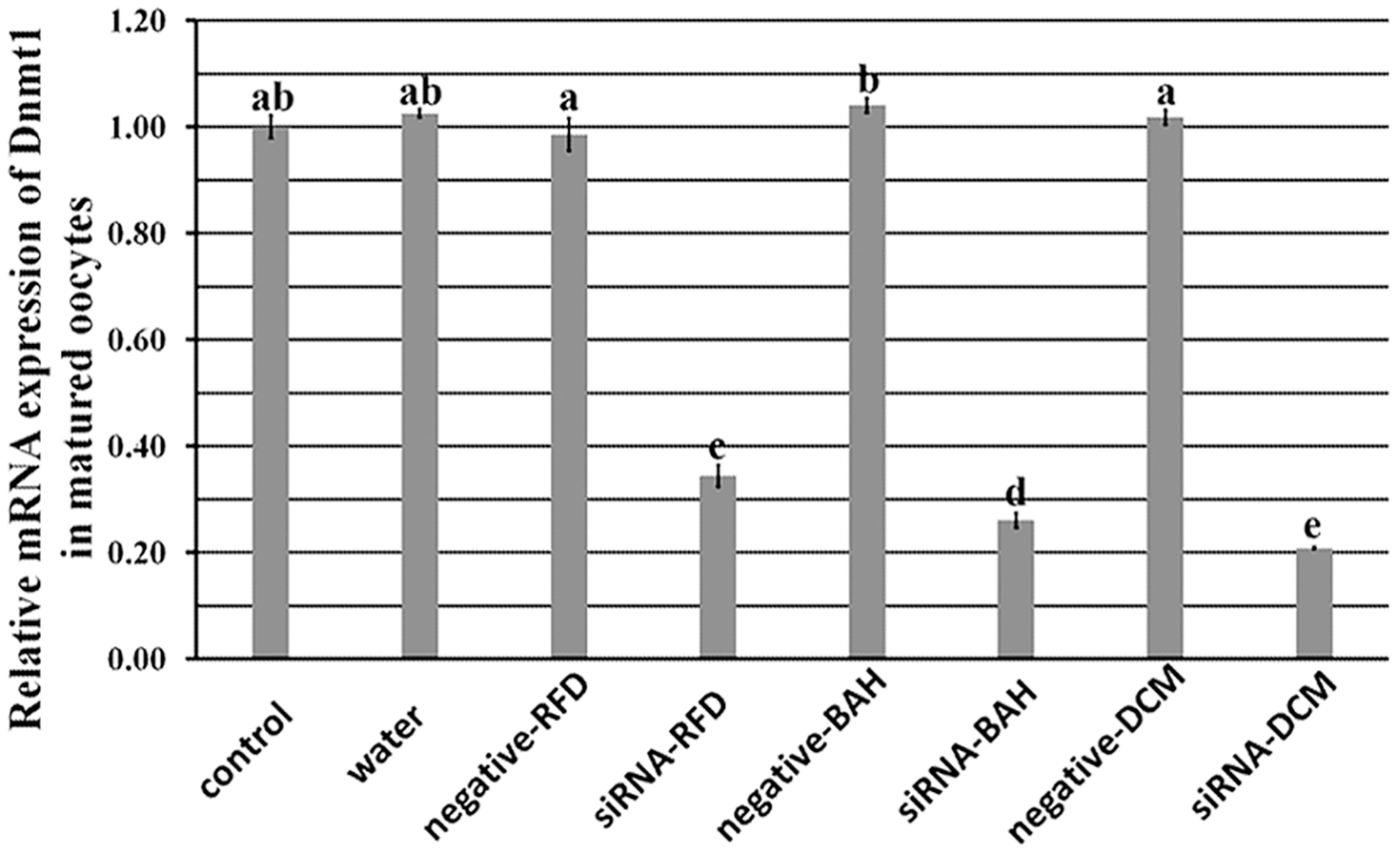
The interference efficiencies among different siRNAs. siRNA-RFD, siRNA targeting the replication foci domain (RFD) of Dnmt1, siRNA-BAH, siRNA targeting the bromo adjacent homology domain (BAH) of Dnmt1, and siRNA-DCM, siRNA targeting the cytosine-C5 specific DNA methylase domain (DCM) of Dnmt1. After siRNAs were injected into GV stage oocytes, the interference efficiencies were mensurated in matured MII stage oocytes. siRNA-RFD, siRNA-BAH and siRNA-DCM significantly reduced the expression of Dnmt1, and the interference level of siRNA-DCM was the highest. ^a-e^Values with different superscripts differed significantly (P<0.05).

When siRNAs were injected into GV stage oocytes, siRNA-RFD, siRNA-BAH or siRNA-DCM resulted in a 66%, 74% or 79% reduction of *Dnmt1* transcripts in matured oocytes, respectively, significantly lower than those in the controls noninjected or injected with water or negative siRNAs (Fig 2, P<0.05). Thus, siRNA-DCM was the most effective interference sequence for *Dnmt1* knockdown in oocytes, and was applied in the following siRNA group.

### Effect of *Dnmt1* knockdown in GV stage oocytes on oocyte maturation

To examine the impact of *Dnmt1* knockdown on oocyte maturation, oocyte nuclear and cytoplasmic maturation were investigated. The results of oocyte nuclear maturation displayed that *Dnmt1* knockdown did not destroy the spindle status, and no significant differences in the rates of the first polar body extrusion were observed between the siRNA and control groups (S2 Fig).

**Fig 3:**
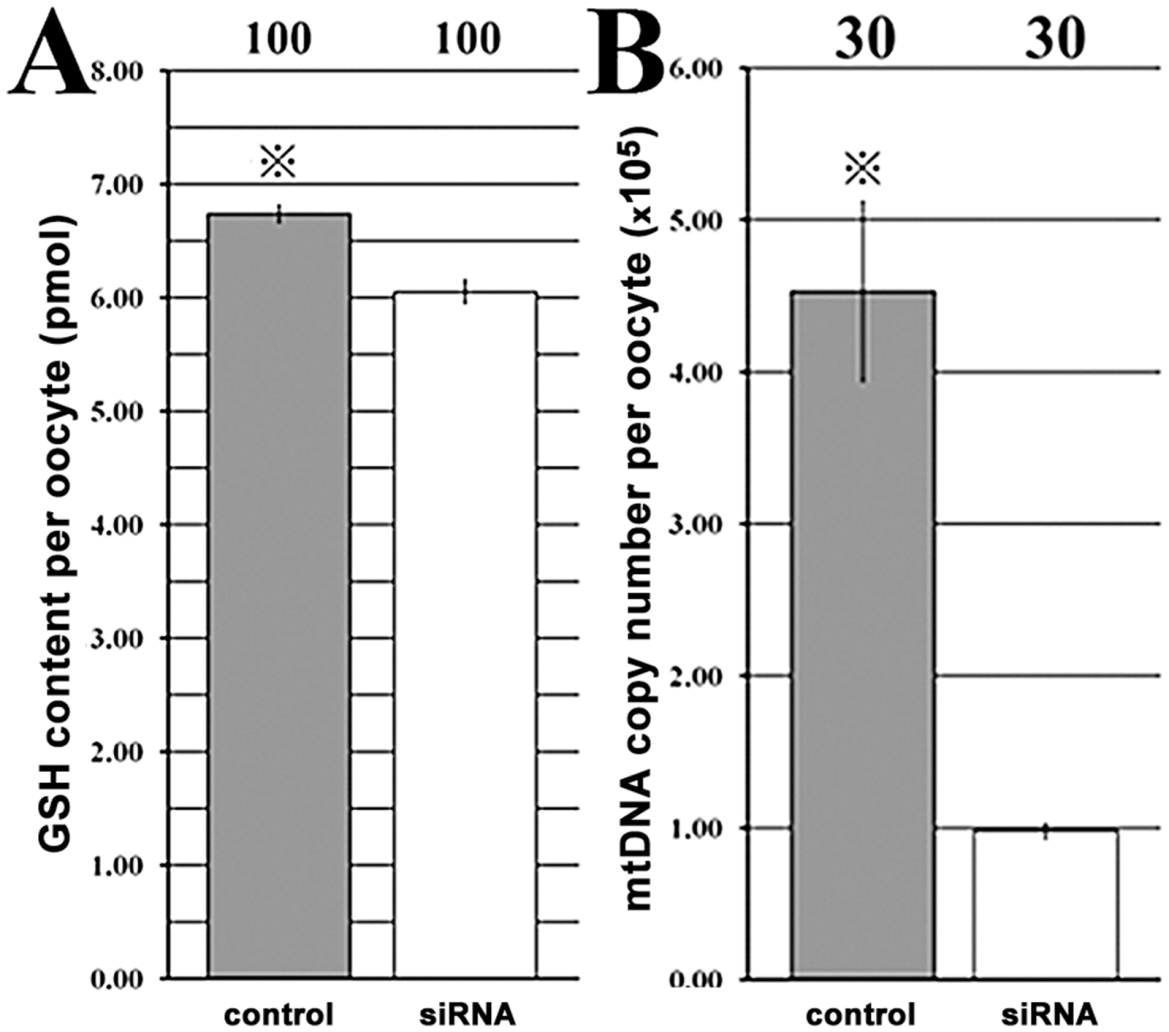
The GSH content and mtDNA copy number per oocyte. A, the GSH content, and B, the mtDNA copy number per matured oocyte after Dnmt1 knockdown. After siRNA injection into GV stage oocytes, the GSH content and mtDNA copy number per oocyte were significantly reduced in MII stage oocytes. The number of oocytes detected was on the top of the column chart, and ^※^Values with a star marker in the same column chart differed significantly (P<0.05).

For oocyte cytoplasmic maturation, the results demonstrated that compared with those in the control group, the GSH content (618.03 vs 1015.63 for the optical density, and 6.05 pmol vs 6.74 pmol for the content, respectively, P<0.05) and mtDNA copy number (862.43 vs 1406.90 for the optical density, and 9.80 × 10^4^ vs 4.53 × 10^5^ for the content, respectively, P<0.05) per oocyte were significantly reduced after siRNA injection (Fig 3 and S3 Fig). And, a significant increase of G6PDH activity, which were negatively correlated with the BCB positive rate (76.29% vs 88.16%, P<0.05), and a significantly destroyed distribution of CGs (62.18% vs 77.54% under the membrane, P<0.05) were also observed in the siRNA group. Thus, *Dnmt1* knockdown did not destroy oocyte nuclear maturation but severely impaired oocyte cytoplasmic maturation.

### *Dnmt1* knockdown disrupted the expression patterns of maternal factors in matured oocytes

**Fig 4:**
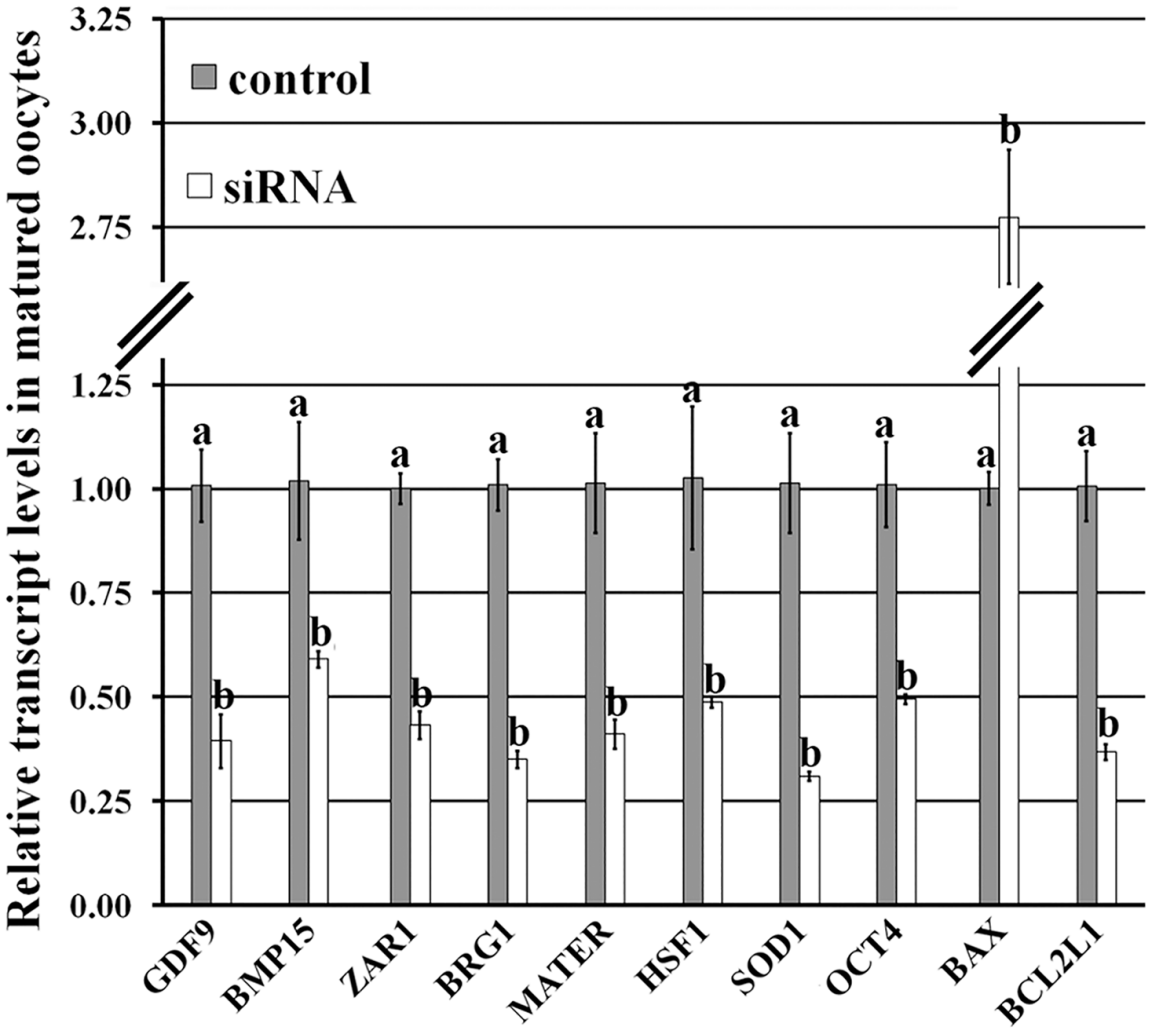
Relative mRNA expression of maternal factors in matured oocytes after Dnmt1 knockdown. After siRNA injection into GV stage oocytes, the transcripts of maternal and apoptosis related genes in MII stage oocytes were examined, and Dnmt1 knockdown significantly reduced the transcripts of Gdf9, Bmp15, Zar1, Brg1, Mater, Hsf1, Oct4, Sod1 and Bcl2l1, but increased the expression of Bax. ^a-b^Values for a given gene with different superscripts differed significantly (P<0.05).

After *Dnmt1* knockdown, the transcription profiles of maternal factors (Fig 4) displayed that the transcripts of oocyte growth (*Gdf9* and *Bmp15*), embryo development (*Zar1*, *Brg1*, *Mater*, *Hsf1* and *Oct4*) and oxidation reduction (*Sod1*) related factors were significantly downregulated in comparison with those in the control group (P<0.05). And more, a significantly increased transcription level of proapoptosis gene (*Bax*) and a significant reduction in the expression of antiapoptosis gene (*Bcl2l1*) were observed in the siRNA group (P<0.05). These results suggest that *Dnmt1* knockdown reduced the transcripts of maternal factors and antiapoptosis gene, and enhanced the expression of proapoptosis gene, thereby resulting in the impaired cytoplasm of matured oocytes.

### *Dnmt1* knockdown in oocytes reduced the development of early embryos

**Fig 5:**
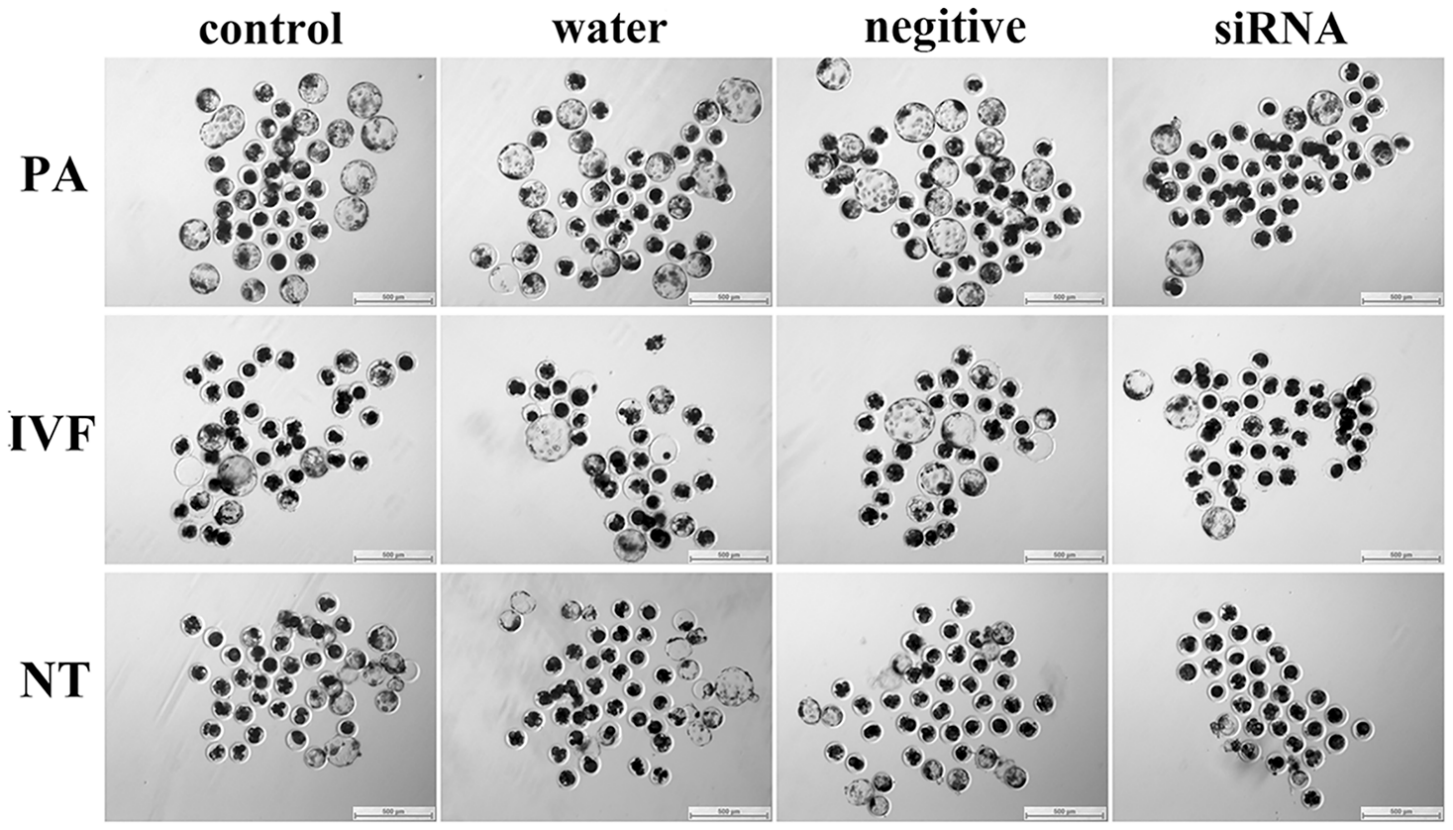
Blastocysts derived from PA, IVF and SCNT after Dnmt1 knockdown. After siRNA injection into GV stage oocytes, the matured oocytes were subjected to PA, IVF and SCNT, and the morphologies of responding blastocysts (×40) were observed at 156 h after embryo culture.

After *Dnmt1* knockdown, these matured oocytes were subject to PA, IVF and SCNT, and the results displayed that the cleavage and blastocyst rates of PA, IVF and SCNT embryos derived from these oocytes were significantly (Fig 5 and Table 1, P<0.05) lower than those in the control groups, and the fusion rate of SCNT embryos was also reduced in the siRNA group. And more, the majority of PA, IVF and SCNT embryos arrested before the 4-cell stage in the siRNA group. Thus, *Dnmt1* knockdown in oocytes reduced the development of PA, IVF and SCNT embryos.

**Table 1:** Development of early embryos after siRNA targeting Dnmt1 injection into GV stage oocytes.

Pattern	Oocyte injection	No. embryos (rep)^※^	No. embryos cleaved (%±SEM)	No. blastocysts (%±SEM)	Blastocyst cell numbers (mean± SEM)^&^
**PA**	control	151(3)	141 (93.36±1.83)^a^	55 (36.49±1.55)^a^	37±3 (n = 55)
	water	144(3)	131 (91.08±1.50)^a^ ^b^	52 (35.99±1.72)^a^	36±2 (n = 51)
	negative	145(3)	134 (92.46±1.76)^a^	51 (35.06±2.32)^a^	37±4 (n = 51)
	siRNA	154(3)	133 (86.39±0.90)^b^	28 (18.22±1.87)^b^	35±2 (n = 26)
**IVF**	control	140(3)	100 (71.19±2.54)^a^	26 (18.56±0.40)^a^	38±2 (n = 25)
	water	141(3)	99 (70.42±1.34)^a^	26 (18.72±1.89)^a^	37±3 (n = 26)
	negative	141(3)	99 (70.18±1.14)^a^	25 (17.83±1.35)^a^	37±2 (n = 25)
	siRNA	159(3)	103 (64.84±1.17)^b^	16 (10.35±1.65)^b^	36±3 (n = 16)
**SCNT**	control	150(3) (68.06±2.46)^a^	132 (88.09±1.59)^a^	30 (20.20±1.64)^a^	36±3 (n = 29)
	water	149(3) (66.22±1.60)^a^ ^b^	131 (87.81±2.25)^a^	27 (18.13±1.16)^a^	35±2 (n = 27)
	negative	149(3) (66.39±2.52)^a^ ^b^	132 (88.55±1.85)^a^	29 (19.44±1.09)^a^	37±3 (n = 28)
	siRNA	157(3) (60.32±1.02)^b^	126 (80.21±1.56)^b^	19 (12.06±1.38)^b^	35±3 (n = 18)

After siRNA injection into GV stage oocytes, the matured oocytes were subjected to PA, IVF and SCNT, then the cleavage (at 48 h) and blastocyst (at 156 h) rates and blastocyst cell numbers of PA, IVF and SCNT embryos were examined, and siRNA injection impaired the development of PA, IVF and SCNT embryos.

^※^Embryos in the NT groups were the fused embryos, and the percentages in the bracket were the fusion rates.

^&^Blastocyst cell numbers of less than 16 were not included.

^a-b^Values in the same column with different superscripts differed significantly (P<0.05).

## Discussion

Oocyte cytoplasmic maturation is essential for embryo development [3]. Here, we demonstrated that *Dnmt1* was required for oocyte cytoplasmic maturation, and *Dnmt1* knockdown in oocytes impaired the subsequent embryonic development.

Previous studies have shown that *Dnmt1* is essential for epigenetic reprogramming and embryo development, and knockout or overexpression of *Dnmt1* leads to loss of genomic methylation or genomic hypermethylation, thereby resulting in embryo lethality [24, 25]. In this study, *Dnmt1* was shown to be present in porcine oocyte cytoplasm, consistent with previous studies [11, 26], and *Dnmt1* expression maintained the high level and did not obviously change during oocyte maturation, suggesting that *Dnmt1*, as a maternal factor, could take a key role in oocyte maturation.

To reveal the role of *Dnmt1* in oocyte maturation, siRNA, a transient manner to turn down or silence gene activity, was applied, comparable to gene knockout [27]. It was observed that all the designed siRNAs effectively downregulated the transcripts of *Dnmt1*, though the interference efficiencies of different siRNAs were various. This may be due to the site and the binding capacity of siRNAs on the target sequence [28]. After siRNA injection, we found that oocyte nuclear maturation was not destroyed as the spindle status and the first polar body extrusion rate were normal, however, oocyte cytoplasmic maturation was severely impaired when GSH content, mtDNA copy number, CG distribution, and gene expression profiles, etc. were adopted to evaluate oocyte cytoplasmic maturation [2, 5]. The reduction of GSH content indicates that *Dnmt1* could have an antioxidant role in oocytes, and the alteration of *Sod1* expression could also prove this potential point [17]. And, the decreased mtDNA copy number and promoted apoptosis reconfirmed the view that *Dnmt1* regulates mitochondrial function and maintains oocyte survival [16, 29]. We also showed that *Dnmt1* was involved in oocyte growth and maturation, as the G6PDH activity and CG distribution were disturbed after *Dnmt1* knockdown. And, the downregulated maternal and reprogramming factors in the siRNA group displayed that *Dnmt1* could regulate gene expression in oocytes [7, 10]. Thus, *Dnmt1* could have a wide-ranging participation in oocyte cytoplasmic maturation and determine the quality of oocyte cytoplasmic maturation.

As maternal factors are essential for the development of early embryos [2, 7], the development of early embryos was decreased after *Dnmt1* knockdown in GV stage oocytes, while the reduced embryo development was not observed when siRNA was injected into MII stage oocytes (Table C in S1 File). And, the expression levels of *Dnmt1* in matured oocytes were positively associated with the development of early embryos (Table D). These results strongly suggest that *Dnmt1* is required for oocyte cytoplasmic maturation, determining the subsequent embryonic development. However, how *Dnmt1* regulates oocyte cytoplasmic maturation related factors to determine the quality of oocyte maturation and the subsequent embryonic development is still unclear, needing further investigation.

In conclusion, our results demonstrated that siRNA could significantly reduce *Dnmt1* expression in porcine oocytes, and *Dnmt1* knockdown resulted in the impaied oocyte cytoplasmic maturation and poor development of early embryos, suggesting a key role for *Dnmt1* in the regulation of oocyte cytoplasmic maturation.

## Supporting Information

S1 Fig Denuded oocyte maturation, siRNA injection and Dnmt1 expression. A (culture at 0 h) and A' (culture at 42 h), the progress of denuded oocyte maturation (×100). The addition of mural granulosa cells maintained the maturation of denuded oocytes. B and B' (successful injection), FITC-labeled nonsilencing siRNA injection into GV stage oocytes (×40). C, relative mRNA expression of Dnmt1 in matured oocytes after FITC labeled nonsilencing siRNA injection. No obvious changes were observed after FITC labeled nonsilencing siRNA injection.
(TIF)

S2 Fig Oocyte nuclear maturation. A, oocyte maturation rate, and B, the spindle status in matured oocytes (×400) after Dnmt1 knockdown. The siRNA group displayed normal maturation rate and spindle status of oocytes.
(TIF)

S3 Fig The GSH content, mitochondrial content, G6PDH activity and CG distribution in matured oocytes after Dnmt1 knockdown. A, the optical density of GSH content per oocyte (×100), B, the optical density of mitochondrial content per oocyte (×100), C, the percentage of BCB positive oocytes (G6PDH activity, ×100), and D, the percentage of CG distribution under the membrane (×400) in matured oocytes after Dnmt1 knockdown. In the siRNA group, significant reductions in GSH content per oocyte, mitochondrial content per oocyte, oocyte BCB positive rate and CG distribution under the membrane were observed. The number of oocytes detected was on the top of column chart, and ^※^Values with a star marker in the same column chart differed significantly (P<0.05).
(TIF)

S1 File The detail content of Tables A-D. Table A, the detail of primers for quantitative real time PCR, Table B, oocyte maturation and PA embryo development after GV stage oocytes injected with FITC labeled nonsilencing siRNA, Table C, the development of PA, IVF and SCNT embryos after siRNA injection into MII stage oocytes, and Table D, the development of PA embryos derived from GV stage oocytes injected with different interference siRNAs.
(PDF)
