# Supplementary figures and images for: A Novel Role for DNA Methyltransferase 1 in Regulating Oocyte Cytoplasmic Maturation in Pigs
Source: PLoS One. 2015 May 26;10(5):e0127512. doi: 10.1371/journal.pone.0127512 (PMC4444208; doi:10.1371/journal.pone.0127512)

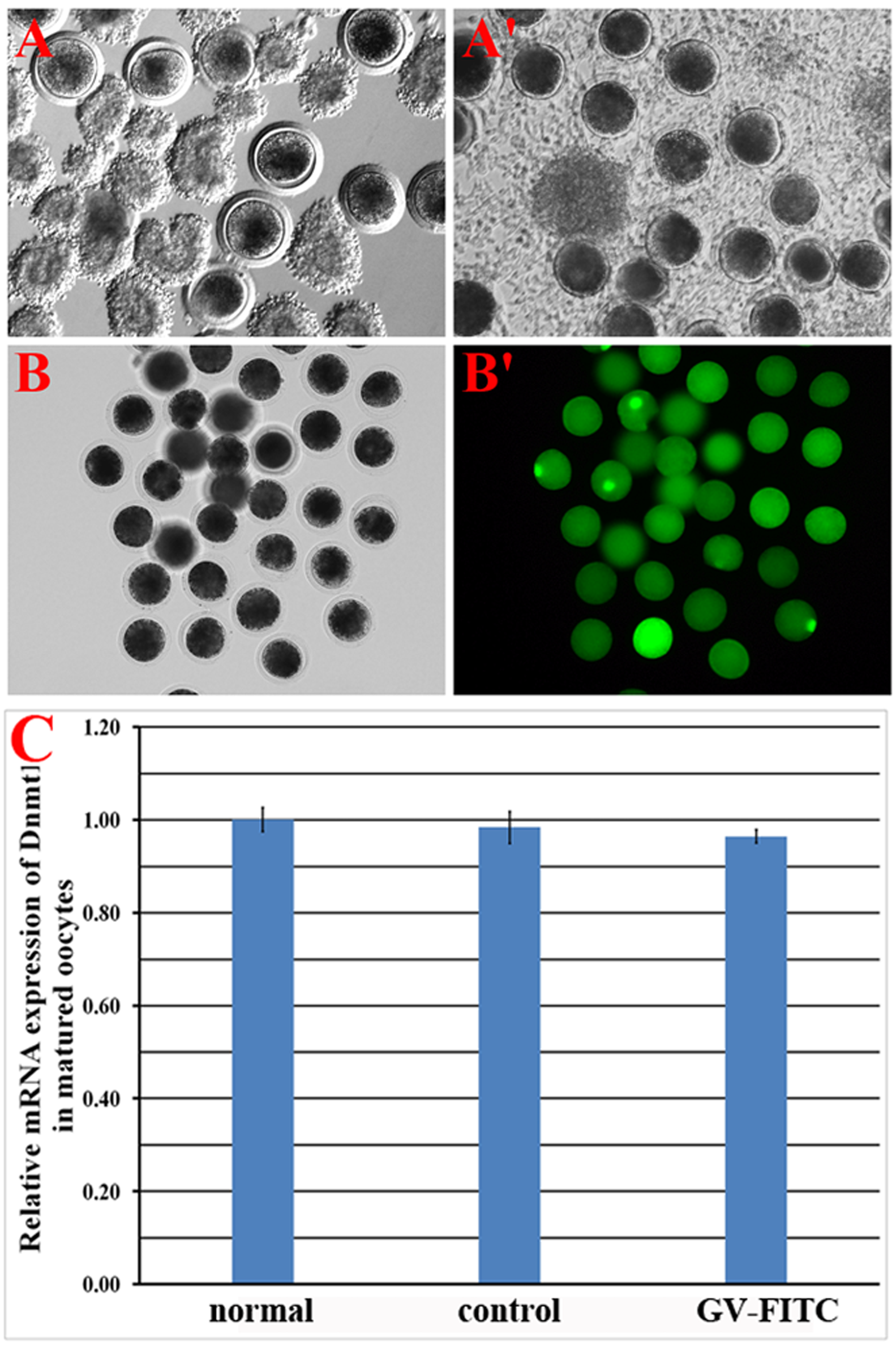

Supplement: S1 Fig — A (culture at 0 h) and A' (culture at 42 h), the progress of denuded oocyte maturation (×100). The addition of mural granulosa cells maintained the maturation of denuded oocytes. B and B' (successful injection), FITC-labeled nonsilencing siRNA injection into GV stage oocytes (×40). C, relative mRNA expression of Dnmt1 in matured oocytes after FITC labeled nonsilencing siRNA injection. No obvious changes were observed after FITC labeled nonsilencing siRNA injection. (TIF) [file pone.0127512.s001.tif]

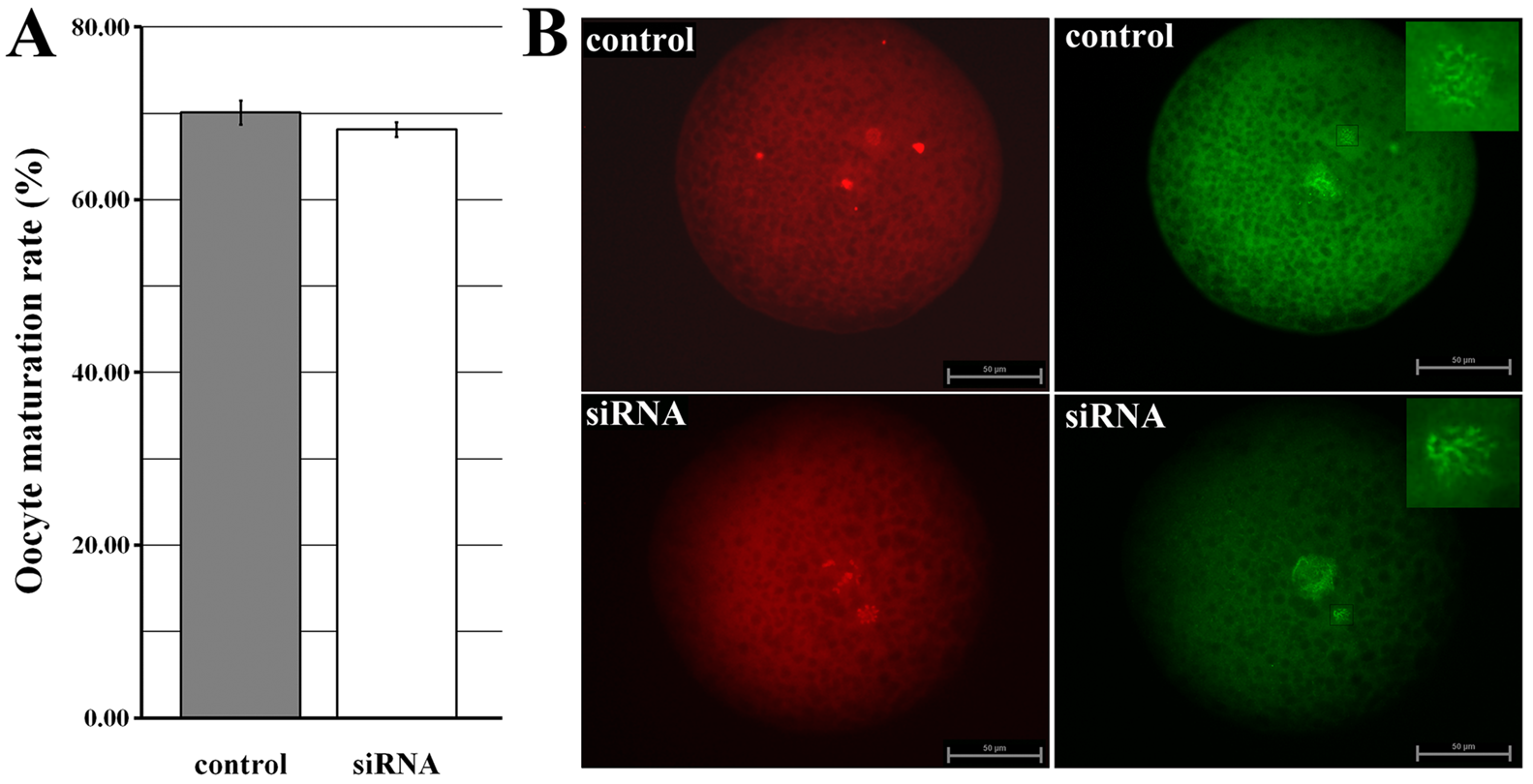

Supplement: S2 Fig — A, oocyte maturation rate, and B, the spindle status in matured oocytes (×400) after Dnmt1 knockdown. The siRNA group displayed normal maturation rate and spindle status of oocytes. (TIF) [file pone.0127512.s002.tif]

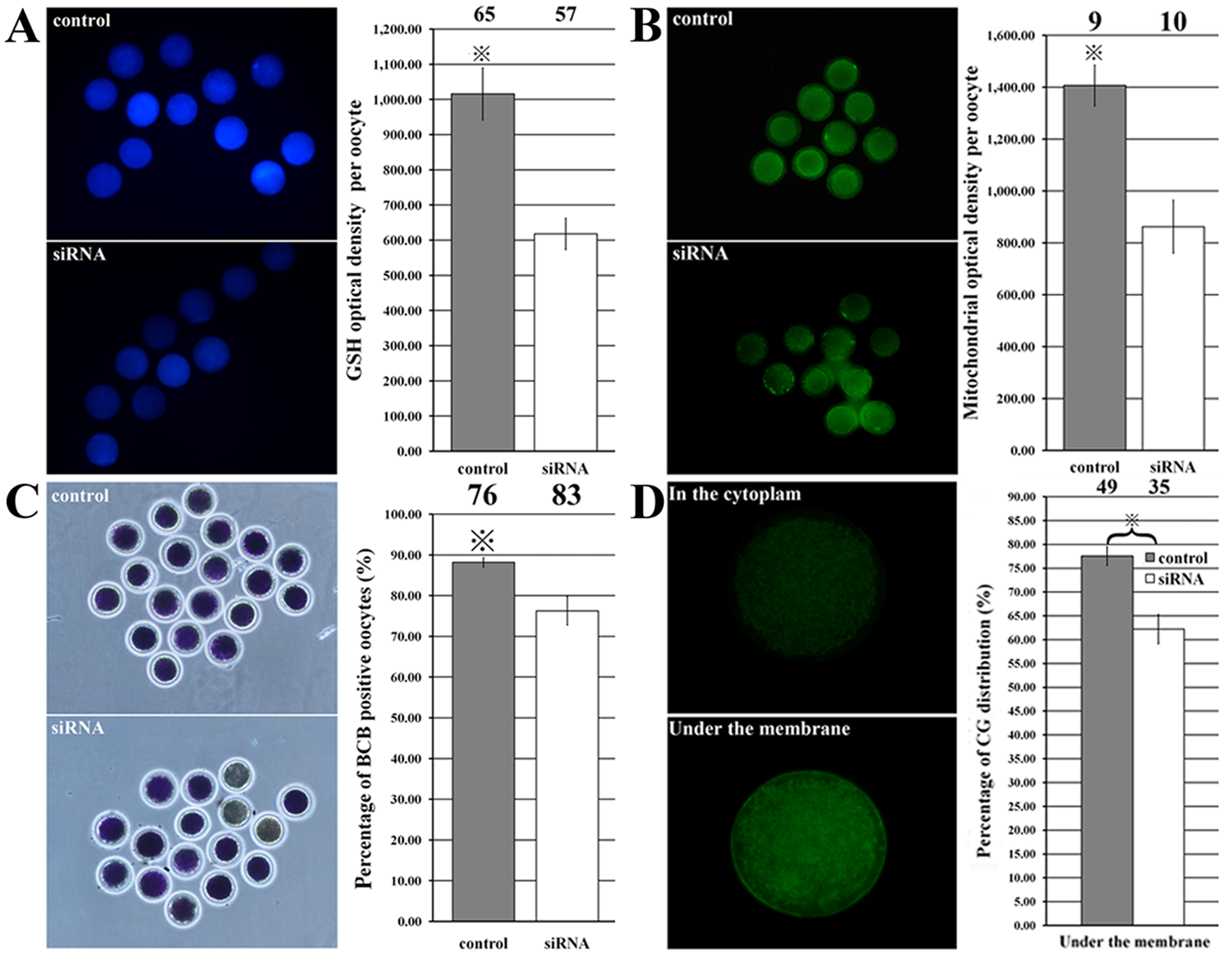

Supplement: S3 Fig — A, the optical density of GSH content per oocyte (×100), B, the optical density of mitochondrial content per oocyte (×100), C, the percentage of BCB positive oocytes (G6PDH activity, ×100), and D, the percentage of CG distribution under the membrane (×400) in matured oocytes after Dnmt1 knockdown. In the siRNA group, significant reductions in GSH content per oocyte, mitochondrial content per oocyte, oocyte BCB positive rate and CG distribution under the membrane were observed. The number of oocytes detected was on the top of column chart, and ※Values with a star marker in the same column chart differed significantly (P<0.05). (TIF) [file pone.0127512.s003.tif]
