# Supplementary material for: A Novel Role for DNA Methyltransferase 1 in Regulating Oocyte Cytoplasmic Maturation in Pigs
Source: PLoS One. 2015 May 26;10(5):e0127512. doi: 10.1371/journal.pone.0127512 (PMC4444208; doi:10.1371/journal.pone.0127512)
Supplement: S1 File — Table A, the detail of primers for quantitative real time PCR, Table B, oocyte maturation and PA embryo development after GV stage oocytes injected with FITC labeled nonsilencing siRNA, Table C, the development of PA, IVF and SCNT embryos after siRNA injection into MII stage oocytes, and Table D, the development of PA embryos derived from GV stage oocytes injected with different interference siRNAs. (PDF) [file pone.0127512.s004.pdf]

Table A in S1 File. PCR primers. The primer sequence, amplified length and gene accession number for quantitative real time PCR.

Table B in S1 File. Oocyte maturation and PA embryo development. The maturation of oocytes and the development of PA embryos derived from GV stage oocytes injected with FITC labeled nonsilencing siRNA were examined, and FITC labeled nonsilencing siRNA injection did not impair oocyte maturation and PA embryo development. The normal group, culture of COCs, the control group, culture of denuded oocytes plus mural granulosa cells, and the GV-FITC group, culture of denuded oocytes injected with FITC labeled nonsilencing siRNA plus mural granulosa cells.

Table C in S1 File. The development of embryos derived from oocytes with Dnmt1 knockdown at MII stage. After siRNA injection into MII stage oocytes, the cleavage (at 48 h) and blastocyst (at 156 h) rates and blastocyst cell numbers of PA, IVF and SCNT embryos derived from these oocytes were examined, and siRNA injection at MII stage did not impair the development of PA, IVF and SCNT embryos.

Table D in S1 File. The development of PA embryos after Dnmt1 knockdown. The interference levels of siRNA-RFD, siRNA-BAH and siRNA-DCM were significantly different. After these interference sequences were injected into GV stage oocytes, and the significantly lower development of PA embryos, along with the obviously higher interference level of Dnmt1, was observed.

Table A. Detail of primers for quantitative real time PCR.

Gene	Primer sequence (5'-3')	Length (bp)	Accession number
**Dnmt1**	F:GCGTCTTGCAGGCTGGTCAGTA<br>R:CTTCTTATCATCGACCACGACGCT	152	NM_001032355
**Gdf9**	F: CAAAGCCAACAGAAGTCACCTC<br>R: GTTCAACAGCAGTAACACGATCC	146	NM_001001909
**Bmp15**	F:CCTGGACACTGCCTTCTTGTTACTCTA TTTC<br>R: TGGTTACTTTCAGGCCCATCGTGCT	195	NM_001005155
**Zar1**	F: CTGCGCTTCCAGTTCTTAGAGC<br>R: CACGCGATAAGGGTTATAAGACTTC	191	NM_001129956
**Brg1**	F: GCTCGGTCTGAACCTCCAATC<br>R: GGTCGACGTTGAGCTTGTACTTG	197	EU_780789
**Mater**	F: GAATGATCGGGAACGTAGCTG<br>R: ACCAGGGAAGTTTAGTACACAAGC	122	AM_941716
**Hsf1**	F: CCCTGAGGAGTGAGGACATAAAG<br>R: CACAGGGCCTCGTTCTCATG	136	NM_001243819
**Sod1**	F: TTGGAGACCTGGGCAATGTGAC<br>R: CTTCCAGCATTTCCCGTCTTTGTA	184	NM_001190422
**Oct4**	F:GAAGGTGTTCAGCCAAACGAC<br>R:CGATACTTGTCCGCTTTC	185	NM_001113060
**Bax**	F: CAGTAACATGGAGCTGCAGAGG<br>R: GCCTTGAGCACCAGTTTACTGG	159	AJ_606301
**Bcl2l1**	F: CTGGTGGTTGACTTTCTCTCCTAC	119	NM_214285

	R: GTTTCCGCTTCTGATTCAGTCC		
**18s**	F:AATCTCGGGTGGCTGAACGC<br>R:CCGTTCTTAGTTGGTGGAGCGAT	143	NR_002170

Table B. Oocyte maturation and PA embryo development after GV stage oocytes injected with FITC labeled nonsilencing siRNA.

Groups	No. oocytes (rep)	No. maturation (mean ± SEM%)	No. embryos	No. cleavage (mean ± SEM%)[※]	No. blastocysts (mean ± SEM%)[※]
**Normal**	234(3)	174 (74.36±0.31)	174	163 (93.42±1.49)	67 (38.45±1.64)
**Control**	440(3)	322 (72.56±1.68)	159[&]	148 (92.97±0.97)	64 (40.33±0.68)
**GV-FITC**	241(3)	176 (72.85±1.35)	175	161 (91.97±0.74)	69 (40.04±2.82)

Note: the normal group, culture of COCs, the control group, culture of denude oocytes plus mural granulosa cells, and the GV-FITC group, culture of denude oocytes injected with FITC labeled nonsilencing siRNA plus mural granulosa cells.

[※]Cleavage and blastocyst rates were adjusted for the cultured embryos.

[&]Matured oocytes were partly used for PA in the control group.

Table C. Development of PA, IVF and SCNT embryos after siRNA injection

into MII stage oocytes.

Pattern	Groups injection	No. embryos (rep)※	No. embryos cleaved (% ± SEM)	No. blastocysts (% ± SEM)	Blastocyst cell numbers (mean ± SEM)&
**PA**	control	151(3)	139 (92.05±1.04)	58 (38.53±1.10)	36±2 (n=57)
	water	145(3)	134 (92.46±1.16)	54 (37.39±2.00)	36±3 (n=54)
	negative	144(3)	133 (92.43±1.12)	53 (36.94±1.94)	35±3 (n=51)
	siRNA	158(3)	145 (91.83±1.09)	55 (34.87±1.72)	36±2 (n=53)
**IVF**	control	147(3)	100 (68.05±1.45)	25 (17.03±0.77)	37±3 (n=25)
	water	144(3)	97 (67.38±1.53)	25 (17.42±0.76)	36±2 (n=24)
	negative	144(3)	98 (67.97±1.06)	25 (17.41±0.86)	37±2 (n=25)
	siRNA	153(3)	102 (66.66±1.96)	25 (16.29±0.90)	37±2 (n=25)
**SCNT**	control	151(3) (70.50±1.69)	128 (84.72±1.20)	27 (17.89±0.47)	37±2 (n=27)
	water	142(3) (67.56±0.44)	122 (86.03±0.93)	26 (18.25±0.68)	36±2 (n=25)

negative	146(3) (67.87±1.10)	124 (84.88±0.93)	22 (17.74±0.13)	35±3 (n=21)
siRNA	155(3) (69.02±1.28)	133 (85.79±0.19)	25 (18.83±0.35)	36±3 (n=25)

※Embryos in the SCNT group were the fused embryos, and the percentages in the bracket were the fusion rates.

&Blastocyst cell numbers of less than 16 were not included.

Table D. Development of PA embryos derived from GV stage oocytes injected with different interference siRNAs.

Groups	No. of embryos (rep)	No. cleavage (mean ± SEM%)	No. blastocysts (mean ± SEM%)
**control**	148 (3)	138 (93.23±0.71)[a]	58 (39.21±1.42)[a]
**siRNA-RFD**	155 (3)	138 (88.99±0.88)[b]	42 (27.16±1.60)[b]
**siRNA-BAH**	159 (3)	140 (88.06± 0.42)[bc]	32 (20.10±0.89)[c]
**siRNA-DCM**	158 (3)	135 (85.45±0.32)[c]	26 (16.43±0.92)[d]

[a-d]Values in the same column with different superscripts differ significantly (P<0.05).
